# Antibiotic tolerance coupled with limited benefit from drainage: Clinical challenges in *Klebsiella pneumoniae* liver abscess

**DOI:** 10.1073/pnas.2606316123

**Published:** 2026-08-03

**Authors:** Zibo Gong, Yawen Guo, Xiwen Liu, Zhe Wang, Guofei Li, Zhihui Chang

**Affiliations:** ^a^Department of Radiology, Shengjing Hospital of China Medical University, Shenyang 110004, China; ^b^Department of Pathology, Shengjing Hospital of China Medical University, Shenyang 110004, China; ^c^Department of Pharmacy, Shengjing Hospital of China Medical University, Shenyang 110004, China

We read with great interest the study by Angeles-Solano et al. (1), which demonstrated that antibiotic tolerance—rather than insufficient drug penetration—drives treatment failure in *Klebsiella pneumoniae* (*K. pneumoniae*) liver abscess (KLA) in a murine model. Building on these findings, we propose to provide complementary clinical data to verify the presence of antibiotic tolerance in KLA and to characterize other key therapeutic challenges. We also describe our clinically relevant rabbit model of KLA.

To provide illustrative support, we deposited relevant clinical data in a public repository (2). The full study protocol was approved by the Research Ethics Committee of Shengjing Hospital of China Medical University (No. 2025PS1255K), and written informed consent was obtained from all participants. These data include paired blood and abscess antibiotic concentrations from patients with KLA who showed limited response to antimicrobial therapy despite subsequent clinical improvement following percutaneous drainage (PD) (Fig. 1*A*). Notably, quantitative analysis showed that antibiotic concentrations in all abscesses achieved the pharmacodynamic target (Fig. 1*B*), although substantial interindividual variability in drug penetration rate, defined as the ratio of antibiotic concentration in abscess to the corresponding concentration in blood, was observed (Fig. 1*C*). These data are consistent with the presence of antibiotic tolerance in KLA. Fortunately, the infections in these patients were rapidly controlled following drainage (Fig. 1 *D* and *E*). We speculate that drainage could reduce intra-abscess pressure and modify the local microenvironment, thus potentially reversing the antibiotic tolerance phenotype of *K. pneumoniae.*

**Fig. 1.**
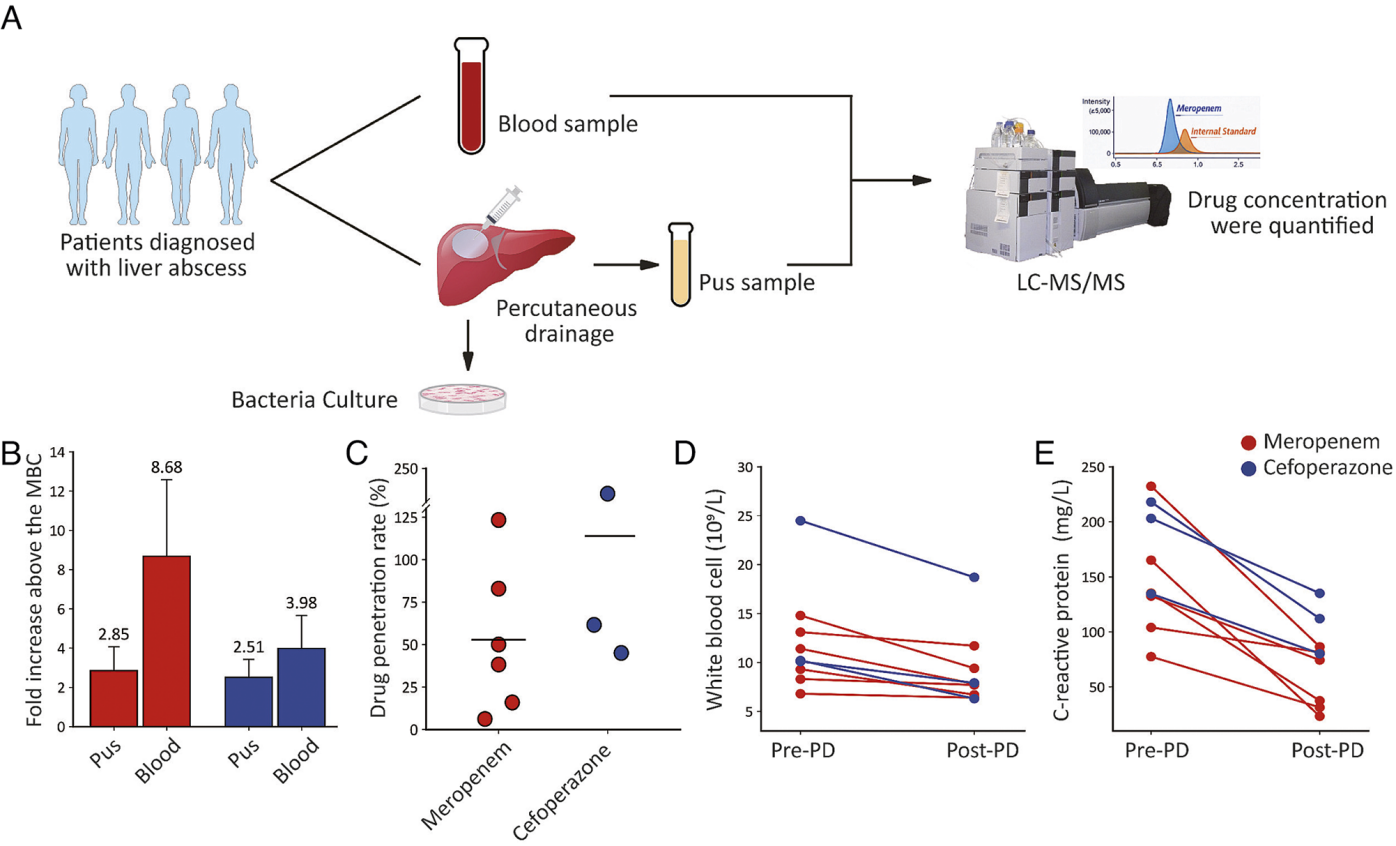
Clinical evaluation of antibiotic tolerance and response to drainage in patients with KLA. (*A*) Workflow. Patients diagnosed with KLA who showed poor clinical response to antibiotic therapy and underwent PD were included. Nine patients had culture-confirmed *K. pneumoniae* infection. Six patients received meropenem and three received cefoperazone–sulbactam. Blood and pus samples were collected at the time of PD, and antibiotic concentrations were quantified. (*B*) Antibiotic concentrations are displayed as a fold increase above the minimum bactericidal concentration (MBC). (Meropenem MBC, 1 µg/mL; Cefoperazone MBC, 16 µg/mL) Bars indicate mean ± SD. (*C*) Interindividual variability in antibiotic penetration rates among patients receiving meropenem or cefoperazone–sulbactam. Horizontal lines indicate group means. White blood cell counts (*D*) and C-reactive protein levels (*E*) within 24 h prior to PD and within 48 h after PD, showing a rapid reduction. Pre-PD: Pre-Percutaneous drainage; Post-PD: Post-Percutaneous drainage.

However, not all patients benefit from PD, particularly those with poor liquefaction or clustered appearance on imaging. The representative patient shown in Fig. 2*A* exhibited minimal clinical improvement despite both antimicrobial therapy and subsequent catheter drainage, from which only a very small volume of fluid could be obtained. To exclude malignancy, a percutaneous biopsy was performed. Hematoxylin and eosin–stained sections revealed extensive necrotic tissue, prominent neutrophilic infiltration, and fibrous hyperplasia (Fig. 2*B*). Even with drainage catheter placement, such lesions often yield very little drainage fluid, and prolonged antibiotic therapy, or even surgical intervention, is still required (3–5). Poor liquefaction and multiple septations not only compromise the efficacy of drainage but may also impede antibiotic penetration into the lesion (6, 7), making these patients the most challenging group in clinical management.

**Fig. 2.**
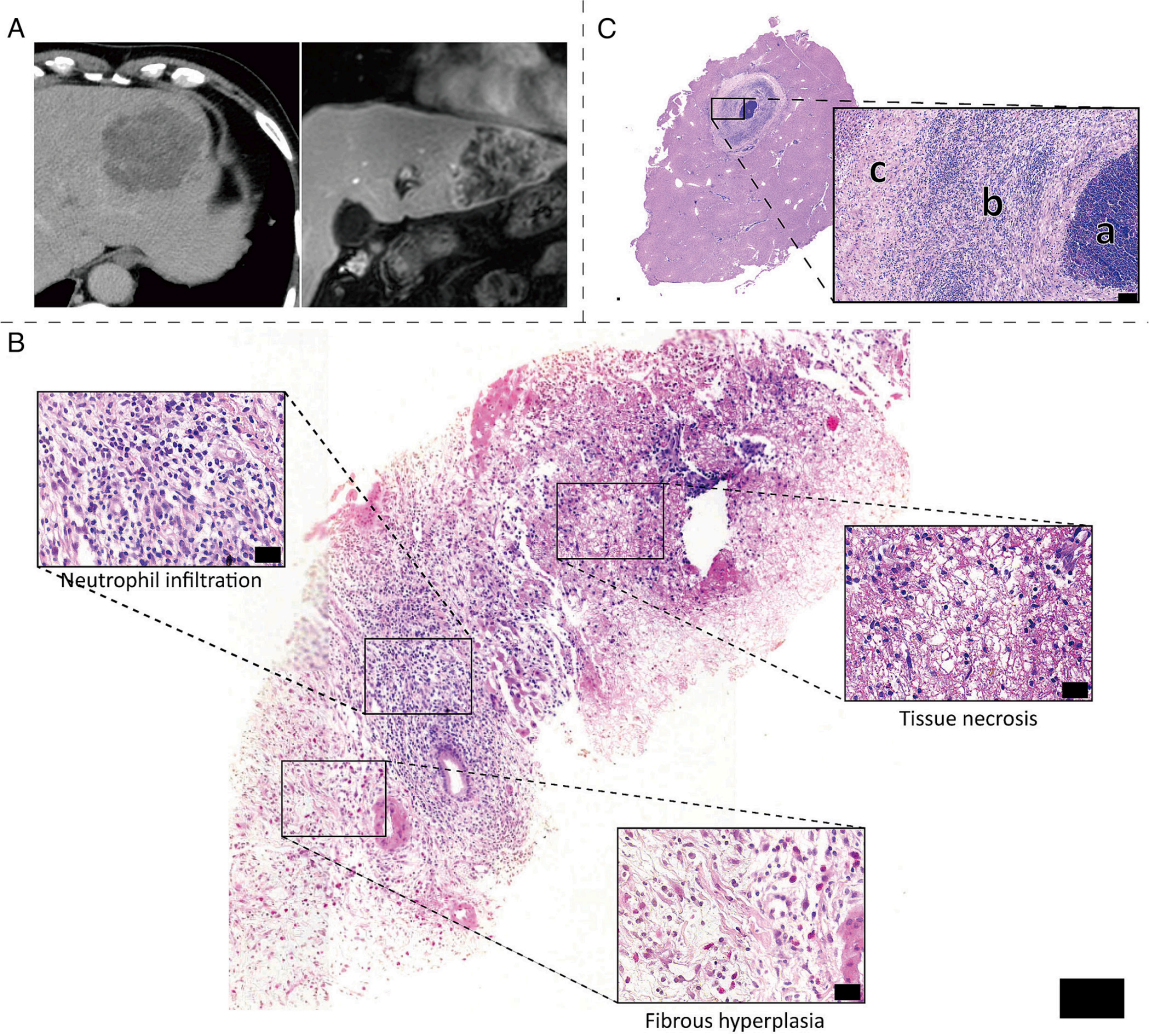
Imaging and histopathological characteristics of a clinical KLA case with limited benefit from drainage and related exploratory animal modeling. (*A*) A 68-y-old male KLA patient. The contrast-enhanced CT (*Left*, venous phase) demonstrated poor liquefaction of the lesion (mean attenuation, 31 Hounsfield units), and contrast-enhanced MR (*Right*) revealed extensive internal septations. (*B*) H&E-stained sections obtained by percutaneous biopsy from the patient revealed extensive necrotic tissue, neutrophilic infiltration, and fibrous hyperplasia. The boxed regions in the whole-tissue section (4×) are shown at higher magnification (10×). (Scale bar, 100 µm.) (*C*) H&E-stained sections of a KLA induced in a New Zealand rabbit by intrahepatic injection of *K. pneumoniae* reveal pathological characteristics closely resembling those observed in the clinical specimen, including a) tissue necrosis, b) neutrophilic infiltration, and c) fibrous hyperplasia. The boxed regions in the whole-tissue section (0.8×) are displayed at higher magnification (10×). (Scale bar, 100 µm.)

Currently, animal models of KLA are most generated in mice and characterized by focal necrosis and microabscess formation (8–10). However, clinical KLA exhibits a fibroproliferative abscess wall and septations, coexisting hepatic tissue necrosis and repair, as well as distinct regions of necrosis, which differ substantially from existing mouse models. In this context, our recent work suggests that rabbit-based KLA models—established via percutaneous direct puncture of the liver with bacterial suspension injection—may better approximate the key structural features of human KLA than murine models, providing a complementary platform for further investigations (Fig. 2*C*).

Furthermore, the development of novel therapeutic strategies for KLA should not only focus on reversing the bacterial antibiotic tolerance phenotype but also target inhibition of excessive fibrous tissue proliferation and promotion of the liquefaction of necrotic tissue, to improve therapeutic outcomes.

## Data Availability

Clinical data have been deposited in Zenodo (https://doi.org/10.5281/zenodo.19344890) (2).
